# Material Model Effect for Simulating a Single-Lap Joint with a Blind Rivet

**DOI:** 10.3390/ma14237236

**Published:** 2021-11-26

**Authors:** Monika Lubas, Arkadiusz Bednarz

**Affiliations:** Department of Aerospace Engineering, Faculty of Mechanical Engineering and Aeronautics, Rzeszow University of Technology, 35-959 Rzeszow, Poland; abednarz@prz.edu.pl

**Keywords:** material model, plastic deformation, blind rivet, numerical analysis, single-lap joint

## Abstract

This paper concerns the influence of the material modeling method on the results of strength analyses. The research object was a single lap joint with a blind rivet (ISO 12996). The results of numerical strength analysis for various configurations of material models with material and contact nonlinearity were compared not only with the experimental results of such a connection but also with the values estimated using classical analytical tools (pressure stress and Hertz stress). The research aimed to determine how the results of numerical analyses (FEMs) were influenced by the method of modeling the material model and how it relates to the experimental results. As part of the analyses, a discrete riveted model and material models were constructed. The analyses took into account various load cases (from 10 to 90% of the connection capacity) to better illustrate the relationship between the numerical and experimental results. As a result of the conducted analyses, it was determined that the linear-elastic model was an acceptable and suggested solution (with a load of up to 90% of the load capacity of the joint connection) for further tests. The work was summarized with general and specific conclusions relating to all cases of numerical modeling. In addition, the summary includes suggestions for future works.

## 1. Introduction

There were many types of mechanical connections. The main factors influencing the selection of the appropriate type of connection depended on the shape of the connected elements, the material, the transferred loads, the execution possibilities, the economic conditions, the method of connection, and the technology of connection. One of the most common types of connection in aviation structures was the riveted connection. The riveted connections themselves can also be divided into blind, solid, semi-tubular, oscar, drive, self-piercing (SFR), and others [1]. Many factors influence the choice of the right type of riveted connection. These factors were directly related to geometrical parameters, working conditions, the technology of joining, preparations before riveting, and, most of all, the type of material of the riveted sheets and the type of rivet material. The largest group of publications that describe the mechanical properties of riveted joints were investigations with solid rivets and classic rivets [2,3,4,5].

Research on riveted joints was discussed both in experimental and numerical research. The authors focus on various geometrical configurations (single-lap and double-lap), as well as various types of connections [6,7,8]. An important aspect related to the load capacity and strength of a riveted joint was the subject of the fatigue strength of such a joint. This topic has already been discussed in the works of Yuan [4] and Neyad [9]. These works used various fatigue models and showed the relationship between the geometry of the joint and the load capacity and low-cycle fatigue of the joint type.

Accurate knowledge of the state of stress and deformation in a joint created by plasticized deformation has a significant impact on the subsequent results of numerical strength analyses. This statement was confirmed in the work of Korbel [3], who performed a numerical FE analysis and experimental analysis of the riveting process. In this work, the author presents how the squeeze force affects the residual stresses around the hole, which affects the fatigue strength. Armentani also observed two types of fracture, through the hole and in the proximity of the hole [10], depending on the level of stress: the higher the applied stress, the more cracking through the hole.

A separate group of articles [2,11] was publicatished on research on riveted joints made with the new mechanical method, self-piercing riveting (SPR). Moraes [2] presented a comparison between experimental and numerical studies of self-pierced riveted joints. The authors simulated the riveting process using a non-linear finite element model and the joint deformation process. Thanks to that, they observed the impact of these processes on the residual stresses and the model of joint failure. Other authors [10,12] carried out fatigue and static strength on cross-shaped SFR joints with aluminum alloy plates at various loading angles. Kam et al. [13] reported the type of effect of die type and joint configuration on mechanical performance, failure mode, and geometrical characteristics on SPR joints of steel and aluminum alloy. The SPR joint between dissimilar material combinations was also experimentally studied by Taek-Eon Jeong and Xiaocong He [14,15].

Comparison of different types of connection, such as hybrid, adhesive, and riveted joints, and their geometrical properties, was found as a subject of many papers. Some authors such as Zaroug [5] and Bula [16] compared the static strength of single lap joints of bolted, bonded, and hybrid joints. In addition, the results were verified with numerical investigations and shear damage models were also used to model the initiation of the damage in the joints. Armentani [17] conducted a numerical analysis of a single-lap hybrid (bonded/bolted) composite joint in tension with different load values and different bolt configurations. Pitta also discussed a wide range of rivet types [18,19]. Pitta [19] also presented an experimental and numerical analysis of repairs on carbon fiber reinforced epoxy (CFRE) substrates, with CFRE and aluminum alloy doublers typical of aircraft structures, repaired with riveted, adhesive-bonded, and hybrid joints. Piątek [20] described that the experimental analysis of anchorages made of steel plates connected to CFRP strips with steel rivets and epoxy adhesive.

All of the authors presented excellent work and insight into riveted joint strength investigations and numerical FEM modeling. However, the influence of the material model on the distribution of stresses and deformations, as well as the influence of other geometric parameters on the load capacity of the riveted joint was not given. From the point of view of this work, the achievements presented in the publication [21] were very important; the authors analyzed in detail the strength of a single-lap joint with a blind rivet and the influence of the hole chamfer size on the joint load capacity. Shear curves proved that the size of the hole chamfer has a great influence on the strength of the joint and the deformation of the rivet during shear.

In papers [22,23,24,25], the authors used elastic-plastic material models for deformable parts of the joint. Presz and Du [22,24] presented various stress distributions in the rivets into the numerical simulation to prove their influence on joining quality. Additionally, Al-Bahkali [25] assessed and reported the thermal stresses developed in riveted joints. In the publications cited above, other types of connections were tested, or other configurations of material models were taken into account. The presented publication fills a gap in the knowledge of the selection of material models for the nonlinear strength analysis of a riveted joint.

In this work, a numerical analysis of single lap blind riveted joints was performed. The aim of the work, the assumption of numerical simulations, was the selection of the material model and modeling of joint and the influence of this model on plastic deformation.

The results of the numerical tests were compared with the experimental results for the selected geometric model of the riveted joint.

## 2. The Object of the Test

### 2.1. The Geometry of Joints and Materials

**Figure 1:**
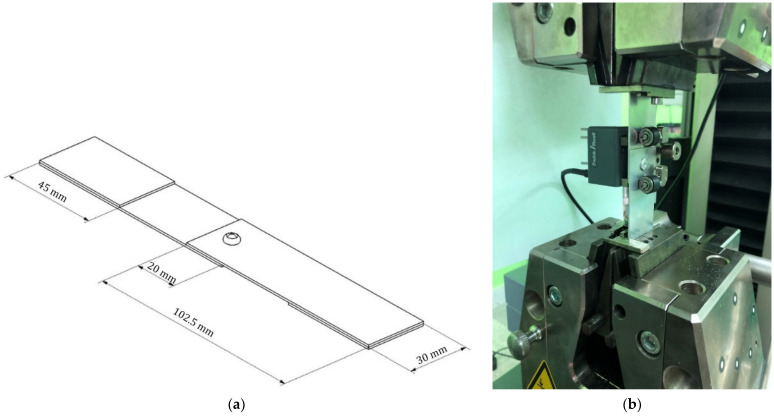
Geometry (**a**) of joints used in numerical/experimental analysis and experimental specimen (**b**) mounted in Endurance Machine.

The object tested in the presented work was a single-lap joint with a blind rivet. The geometry of the riveted joint was shown in Figure 1a. Dimensions were taken from standard ISO 12996 [26]. The sheets were made of EN AW 2017A aluminum alloy with a thickness of 1 mm and the blind rivets were made of EN AW 5251 aluminum alloy with a diameter of 4 mm. According to the standard [26], additional plates should be glued to the ends of the joined sheets to ensure the axial nature of the load (as seen in Figure 1a). The mechanical properties of the sheet material and the blind rivet material were given in Table 1.

The choice of material was based on commercially available rivets and one of the most popular sheets used in the aviation industry. The strength values presented in Table 1 have been obtained from the available literature [27,28] and have been experimentally verified in the Material Strength Laboratory of the Department of Aerospace Engineering at the Rzeszow University of Technology.

### 2.2. Tension Test of the Joint

The experimental tests were carried out on a Zwick–Roll tension machine (Figure 1b) equipped with an extensometer and a force transducer with a nominal force value of 50 kN. According to the ISO 12996 guideline, all experimental tests have been carried out under a constant for the traverse speed of 4 mm/min.

**Figure 2:**
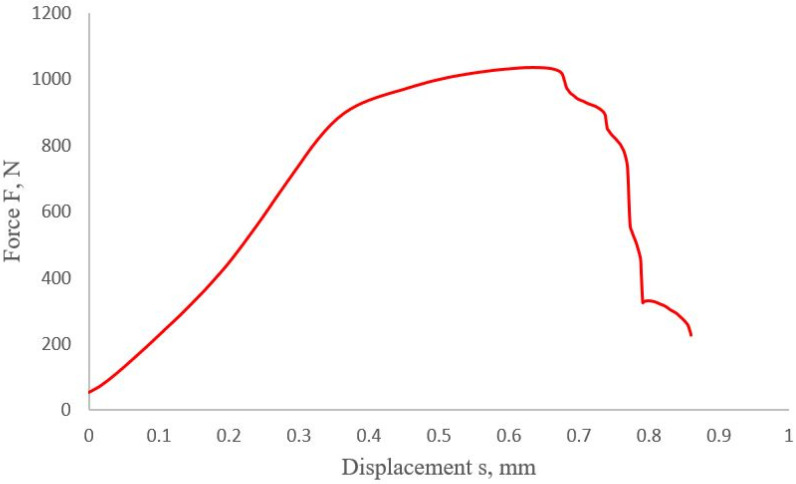
The shear curve for the joint connection was examined.

During the joint shear test, the actual force F (with a measurement accuracy of 0.12% of the nominal force value) and the sheet displacement s (measurement uncertainty 0.5 μm) were monitored and recorded. Thanks to the static tensile test of the riveted joint, it was possible to prepare a diagram for a given type of joint. The graph obtained will be used in later numerical studies, taking into account various numerical models of the material. In the investigation itself, 10 samples were tested, and, using the arithmetic mean, a representative graph of the static tensile test was determined, presented in Figure 2. The graph starts with the value of 50 N because this value of the force was set as the pre-stress, erasing clearances in the locks of the testing machine.

First, the sample was mounted in the jaw chuck of the testing machine and loaded with preload in order to remove play in the clamps of the chucks. Then, the extensometer was mounted (Figure 1b) to increase the accuracy of the deformation reading in the area of the rivet joint. The test was continued and the extensometer was removed at a load of about 500 N (protection against damage to the measuring system). In the further part of the test, the deformation was read from the displacement of the traverse and the assumed length of the sample. After the sample was broken, the traverse was stopped and the separated elements of the riveted joint were taken out.

As a result of the load capacity test (tensile test), the maximum value of the transmitted force was determined at the level of 1036 N (with an elongation of 0.635 mm) (Figure 2). Breaking of the joint was achieved with the traverse path value of s = 0.856 mm. Based on the ISO 12996 [26] standard, taking into account the safety factor, the force in the connection was assumed to be 0.3Fmax, where Fmax was the maximum force transferred by the connection (1036 N). Hence, 0.3Fmax = 311 N.

The presented curve will be used in later numerical studies as a reference/comparison point for numerical analyses, taking into account different methods of material modeling (linear and nonlinear materials). The most important aspect, from the point of view of engineering calculations, was to match the results in the range from 0–90% of the maximum force (connected to UTS) transmitted by the riveted joint.

## 3. Analysis of Contact Stress

Before starting the preparation of the numerical strength analysis, performed using the finite element method, a preliminary stress analysis in the riveted joint was performed using Hertz stresses. At the point of contact of two elastic bodies, pressed against each other by a certain force, stress appears in a small area of contact between the bodies, which was called contact stress.

For analytical calculations of contact stresses, the following simplifying assumptions were made (following Hertz stress theory) [29]:

- The deformations were small and were within the elastic limit (the materials were subject to Hooke’s law);
- The bodies were limited by smooth surfaces of regular curvature;
- The contact area was much smaller than the characteristic dimensions of the bodies in contact;
- Each body can be considered a flexible half-space;
- Only normal stresses occur on the contact surface;
- The surfaces were friction-free.

**Figure 3:**
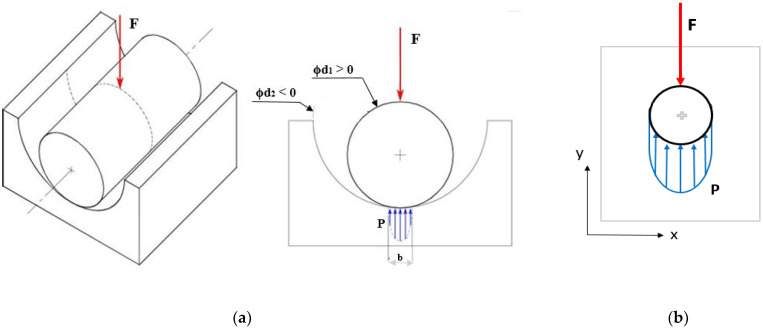
(**a**) Geometric definition of the Hertz problem; (**b**) stress between two objects in contact (without clearance).

The values of contact stresses for the shear of the rivet were consistent with Hertz’s theory for the contact of two surfaces (concave and convex). The graphic definition was presented in Figure 3a. The most important values used in the calculations of stresses according to Hertz were: 

F—force transmitted by elements, N; 

$d_{1}$—diameter of the negative (smaller object), mm; 

$d_{2}$—diameter of the larger object, mm.

For calculations, the relative diameter of the curvature ($d_{*}$) should be determined by the Formula (1):

(1) $$\frac{1}{d_{*}}=\frac{1}{d_{1}}+\frac{1}{d_{2}}$$

An equivalent module ($E_{*}$) of elasticity for both materials of cylindrical connection was (2):

(2) $$\frac{1}{E_{*}}=\frac{1-\nu_{1}^{2}}{E_{1}}+\frac{1-\nu_{2}^{2}}{E_{2}}$$

where:

$E_{1}$—Young’s Modulus of the material of part 1;
$E_{2}$—Young’s Modulus of the material of part 2;
$\nu_{1}$—the Poisson’s ratio of the material of part 1;
$\nu_{2}$—the Poisson’s ratio of the material of part 2.

The width of the contact surface for a cylinder–cup contact (parameter *b*) was defined by Equation (3). The equation for the width of the joint depends on the force, the contact length (*L* = 1 mm), and the relative diameter of the joint ($d_{*}$), as well as the relative Young’s Modulus ($E_{*}$). Theorem-type environments (including propositions, lemmas, corollaries, etc.) can be formatted as follows:

(3) b=(2FπL·d∗E∗)12

Based on the width of the joint (indent width), it was possible to determine the maximum value of the stresses. This value was expressed in Equation (4):

(4) $$P_{max}=\frac{2F}{\pi bL}=\sqrt{\frac{2FE_{*}}{\pi Ld_{*}}}$$

In the case of the analyzed riveted joint, the diameter dimensions were experimentally estimated (using an optical microscope). The values of the diameters $d_{1}$ and $d_{2}\;$ were 4.09 mm and 4.1 mm. After substituting the above quantities into Equation (1), we obtain $d_{*}$ = 0.5562 mm.

In turn, substituting the material data from Table 1 (Young’s modulus and Poisson’s ratio), remembering that element 1 was a rivet and element 2 was a sheet (using Equation (2)), we obtain the following relative Young Modulus $E_{*}$ = 38.43 GPa.

To verify the values obtained, simple strength calculations, typical of a riveted joint, were performed (graphical definition presented in Figure 3b). These calculations were made based on the assumption of no clearance (only pressure stresses). Such stresses were calculated based on Formula (5). The results of the computational stresses for the analyzed loading force values were presented in Table 2.

(5) $$P_{max*}=\frac{F}{d_{1}L}$$

As a result of the analytical calculations, the stresses values (Hertz and pressure) were determined depending on the applied force for the analyzed rivet joint. It can be observed that, for the small force value (100 N), the Hertz contact stresses were about 45 times higher than the contact stresses (Hertzian stress was almost 1091 MPa). However, a further increase in the force value causes a significant increase in the difference between the two methods. For forces above 600 N, Hertzian stress was about 2500 MPa higher than values of pressure. Interestingly, the force of 900 N, for the method of calculating the pressure stresses, caused the contact stress to exceed the UTS for both materials (sheet and rivet). Calculated Hertz stress values significantly exceed the tensile strength for the tested objects. Due to the above, Hertzian stresses will not be used in further calculations and analyses.

## 4. Numerical Analysis

### 4.1. Discrete Model and Assumptions

Numerical analysis was performed on single-lap joint configurations using 3D finite element models created and analyzed in commercial finite element code ANSYS. The geometrical model used in the numerical analysis corresponded to the riveted joints of the experimental work (Figure 1b).

Three material configurations will be tested in the work. In the first case, only linear elastic material models were used. In the second configuration, the cooperation of the linear-elastic (sheet) and elastic-plastic (bilinear for the rivet) model was investigated. In the last research configuration, two nonlinear (elastic-plastic) models were used.

**Figure 4:**
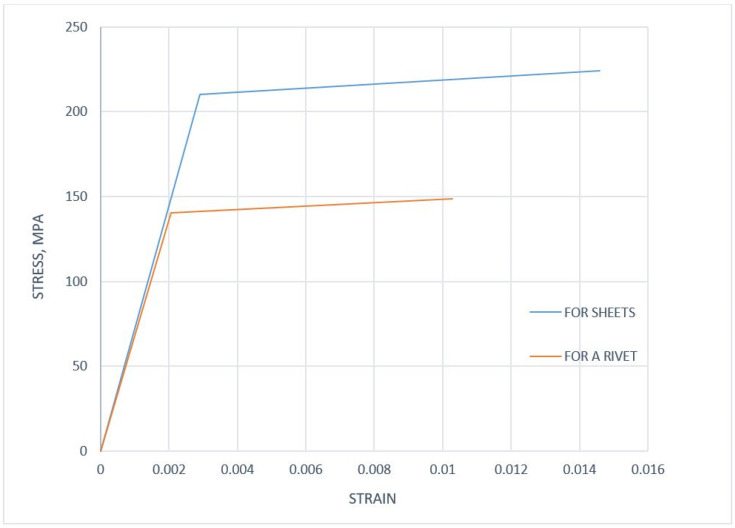
Stress–strain curve of the bilinear material model for sheets and a rivet.

Models of elasto-plastic materials were considered. The true stress–strain curves of the bilinear material model for a sheet and a blind rivet were shown in Figure 4. The data on material properties used to define the material models are presented in Table 3.

The numerical analysis presented was based on several assumptions. These assumptions were made to reflect the state of load obtained in the experimental research discussed earlier. The correct mapping of the state of axial load generated during the static tensile test was crucial to obtain a satisfactory agreement of the experimental and numerical results. The geometric model used in further analyses was the same as in the case of experimental research (Figure 1a). As a result of the evaluation of the experimental data, the following boundary conditions of the numerical strength analysis (based on the FEM method) were established.

The mechanical boundary conditions used during the tests were as follows:

- The right end of the sheet and the right additional surface (fixed support—all degrees of freedom were fixed);
- The end of the sheet and left additional surface (allowed displacement: x = free, y = 0, z = 0);
- The left end the external load (force F, N) acting in the x-direction;

Moreover, to reflect the state of cooperation of elements (sheet and rivet), the following contact conditions were adopted:

- sheet-to-sheet frictionless contact;
- sheet(hole)-to-rivet—rough contact.

The selected contact models were selected on the basis of a series of simulations showing the actual separation of the cooperating elements (without penetration). Similar contact conditions have been defined in the references cited earlier [8].

**Figure 5:**
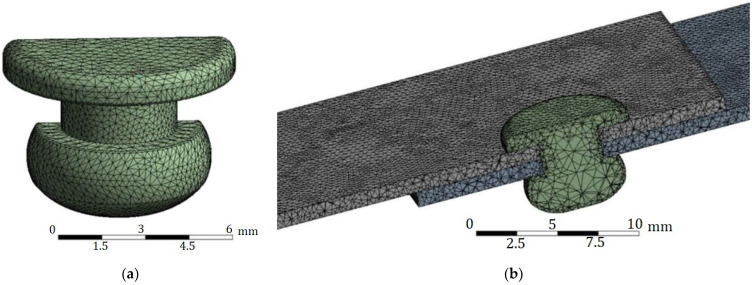
Mesh: (**a**) rivet; (**b**) riveted joint (cross section).

The geometrical (discrete) model consists of three solids: a blind rivet and two sheets, whereas the FE mesh contains more than 900,000 elements (tetrahedral elements with 10 nodes) with a quadratic square function (Figure 5b). Figure 5a shows the mesh of the rivet (after the riveting process—not conducted during the numerical tests presented in this document). To obtain more accurate results in the area of the rivet (more precisely, the rivet mandrel), additional compaction of the elements was assumed on the contact surfaces. The size of the elements and their density was selected through a series of numerical analyses aimed at obtaining the convergence of the results.

The presented discrete model was loaded with the aforementioned loads and contacts. The results of the above-mentioned numerical strength analysis are presented later in the work.

### 4.2. Results of the Numerical Analysis

As part of the numerical analysis of a rivet connection under tension by an axial force, several forces were selected that will load the analyzed object. The maximum load capacity of the tested joint was 1036 N, therefore the tests took into account the forces of 100 to 900 N (approximately 80–90% of the total strength of the joint).

As in the case of analytical calculations of contact stresses, as part of numerical analyses, calculations for forces were carried out: 100, 200, 600, 800, and 900 N. Detailed results for all analyses were presented in Appendix A. The above-mentioned annex includes the value of the force and total elongation (from the experiment), as well as the following results of numerical analyses: total elongation, the maximum value of equivalent stress (von Mises), maximum total displacement for the rivet only, maximum equivalent stress (von Mises) for the rivet, the maximum value of shear stresses, and their minimum and average value. Only the stress distributions for one force (900 N) case will be discussed in the publication.

**Figure 6:**
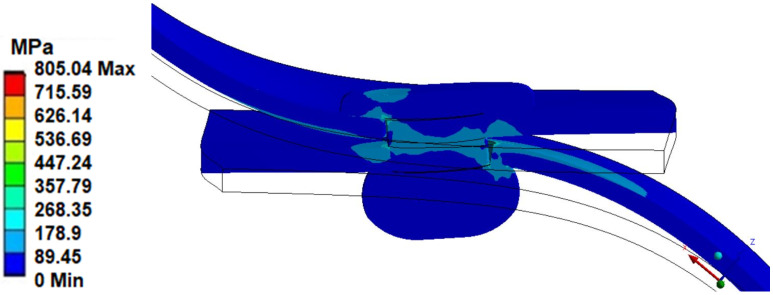
The equivalent stress of the joint connection.

In the case of numerical tests carried out for a riveted joint loaded with an axial force of 900 N, the highest value of equivalent stresses was obtained for the edge of the sheet in contact with the rivet shank (value 805 MPa—linear elastic model). Stress concentration was observed around the site of occurrence (Figure 6). An additional stress concentration, following the load diagram, was observed in the area of the plates’ pressure on both ends of the rivet (this results from the gentle rotation of the plates due to the elimination of clearances).

**Figure 7:**
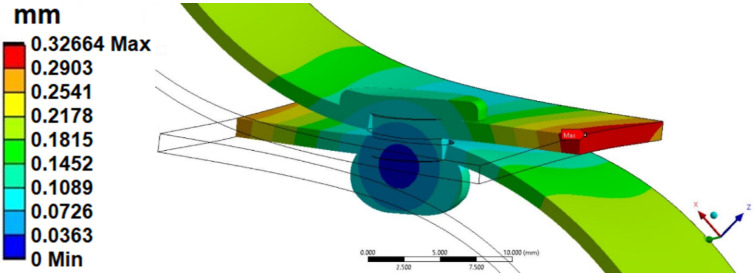
Total deformation of the joint connection of the rivet.

**Figure 8:**
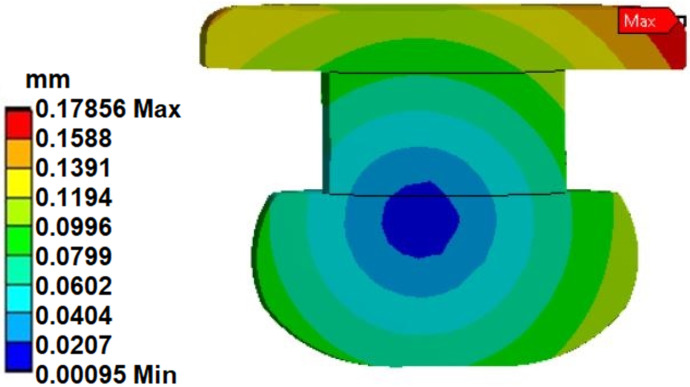
Total deformation of the rivet.

Maximum displacement (0.32 mm—Figure 7) was observed at the edge of the plate. This displacement was both a radial and an axial displacement. The end of the sheet moves by a comparable amount. In the case of the analysis of the displacements of the rivets themselves, their rotation can be observed (Figure 8), where the pivot point was the area of contact of the rivet head with the plate (blue center point in Figure 8). This rotation, together with the deflection of the upper part of the rivet, locates the total displacements in the upper right corner of the rivet section (Figure 8). The value of the total displacement observed was close to 0.18 mm (with the force F = 900 N, linear elastic models).

**Figure 9:**
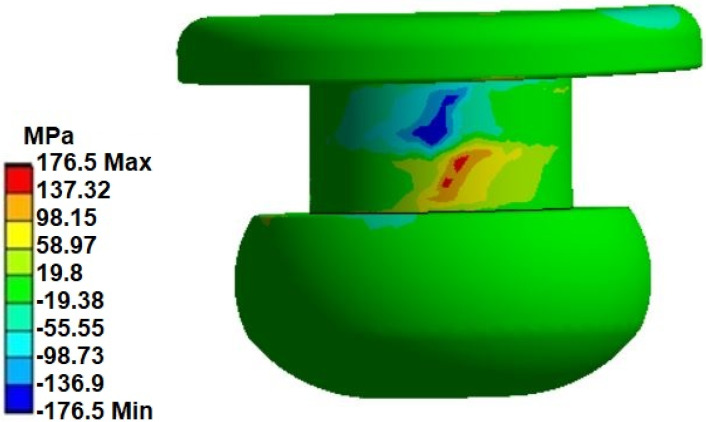
Shear stress (XY plan) on the rivet.

Moving on to the analysis of the stress state in the rivet itself (in the case of F = 900 N, linear models), the values of the shear stresses in the XY plane should be discussed (Figure 9). the maximum value of shear stresses was 176 MPa, while the lowest was −176 MPa. These values appear on the sides of the rivet (they were the result of the pressure of the walls of the change in the shape of the hole in the plate).

**Figure 10:**
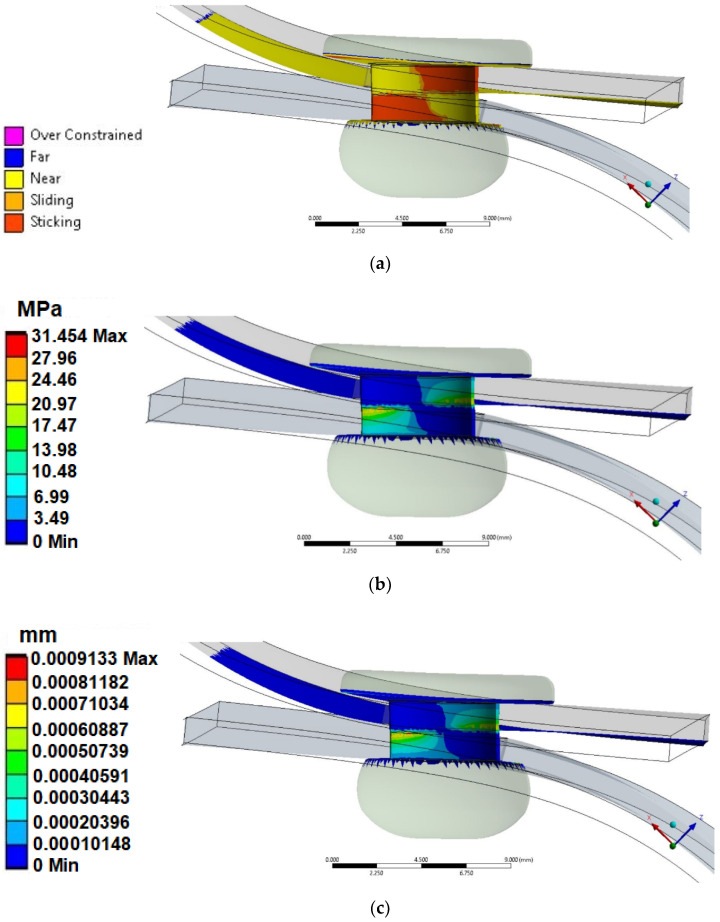
(**a**) Contact status; (**b**) contact pressure; (**c**) penetration.

To verify the correct operation of the riveted joint, the analysis used a tool to verify the contact state (distinguishing between glued/sliding/close, far, and oversized bodies). This algorithm was based on the mutual constraints between the surfaces and their real distance between them. On the mandrel of the rivet (Figure 10a), the contact is observed with the sheets, sliding on the sides, and “close” on the rest of the rivet surface.

Another tool used in the assessment of the working condition of a riveted joint was the tool to assess the pressure between the working surfaces. Using the pressure tool, a value of the maximum contact stress of approximately 31.5 MPa was determined (Figure 10b). The maximum values were oriented halfway around the connection. This was due to the fact that, at this point, there was contact with the plates in tension that was directly related to the gentle rotation of the joint, which causes greater pressure on the edges of the plate.

The last but not least important result obtained in the “Contact Tool” was information on the level of mutual interference/penetration between the cooperating bodies. The maximum penetration (Figure 10c) was observed at the same location where the highest values of contact/pressure stresses were detected.

**Figure 11:**
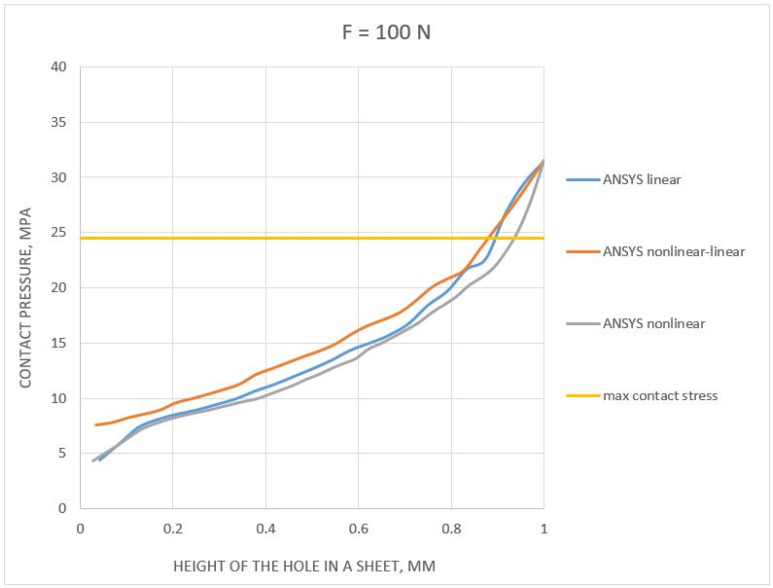
Pressure distribution in contact between the sheet and the skank of the rivet (for a load force F = 100 N).

**Figure 12:**
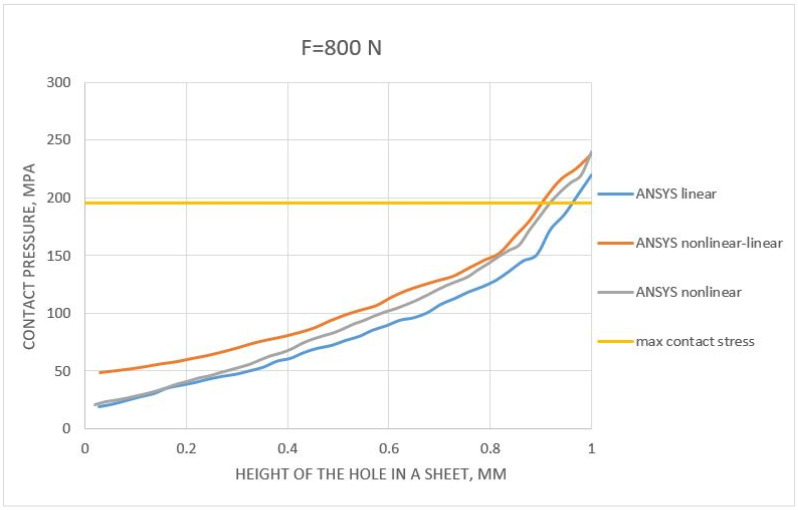
Pressure distribution in contact between the sheet and the skank of the rivet (for a load force F = 800 N).

The next step in evaluating the results of the numerical tensile analysis of the unilateral rivet joint was to determine the contact stresses along with the height of the rivet shank (from 0 to 1 mm, where 0 corresponds to the location at the edge of the rivet and 1 mm corresponds to the center of the rivet, which was half its height). The distributions obtained for all configurations of material models, including the calculated contact stresses, were presented in Figure 11 and Figure 12 (for the forces 100 and 800 N, respectively).

For a force of 100 N, the contact stresses were between 4 and 8 MPa in the area of the edge of the rivet (Figure 11) and, in the peak, it was about 32 MPa (for all material configurations). For comparison, the pressure stress reached a value of about 25 MPa (less than the maximum value from all analyses). It follows that the contact pressure stresses underestimate this value.

The very course of the contact stress values along the rivet height was similar for all material models, although the courses between them have significant deviations ± 5 MPa). The maximum value of 32 MPa for all cases analyzed was expected due to the fact that the loading force should not generate a plastic deformation (works in the elastic range, therefore the results were similar).

Analyzing the load case with a force of 800 N (Figure 12), the contact stresses were about 220 MPa. Unfortunately, the estimated value underestimated the maximum numerical contact stress value (below the maximum value obtained in the FEM numerical analyses). The stress distribution along the rivet height itself was similar to the 100 N force. However, a difference in the maximum values obtained in half of the rivet height (1 mm) was observed. Half of the rivet height was the same distance as the height of the hole in the sheet. Both nonlinear analyses gave similar results (about 240 MPa), while the result from the linear analysis was about 15 MPa less (i.e., about 215 MPa). However, this does not change the fact that all analyses obtained a maximum value higher than that determined by analytical methods. The error itself (between numerical analyses) was around 5%. An interesting observation was the fact that the analysis based on the nonlinear–nonlinear set (both bilinear models), in the area of the edge of the rivet, gave similar values to the analysis based on the linear models, while, in the area of the rivet center (1 mm), the results were similar to the nonlinear–linear analysis (sheet modeled with a linear model and a bilinear rivet).

## 5. Comparison of Results

**Figure 13:**
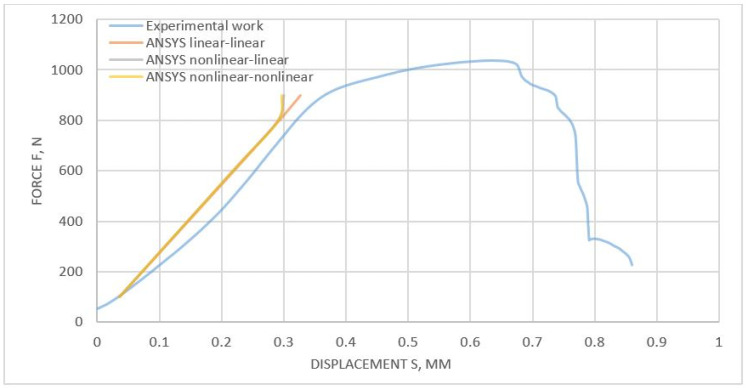
Experimental and numerical load-displacement curves for the riveted joint.

To determine the global impact of the selection of material models on the results of the static tensile test of a riveted joint, it was necessary to compare the experimental and numerical results. For this purpose, the numerical results (presented in Appendix A) were plotted on the graph of the static tensile test (Figure 2), and the calculated displacement (Figure 13) was marked for the given force values. As a result of the graphical comparison of the tensile plots, it can be seen that, in a range up to about 800 N, no differences can be observed between the sets of material models. In the case of a further increase in the loading force, a deviation in the obtained displacements towards lower values can be observed. Therefore, it can be concluded that the best results were obtained for a set of linear models.

Based on the results presented in Figure 11, for a load of 100 N, a 6.27% difference was observed between the linear material model compared to the experimentally obtained result. The difference (5.98%) was observed for nonlinear–linear and nonlinear material models between the numerical and experimental results. The difference/error for the 900 N load was 10.96% for the linear model, 18.07% for the nonlinear–linear model, and 19.11% for the nonlinear model material in comparison to the experiment.

Table 4 presents the experimental and numerical values of the total deformations for the applied loads. For the numerical models, the total deformation maximum was higher by an average of 6.08% (for F = 100 N) and was lower by an average of 16.05% (for F = 900 N) than the experimental results of deformations. It can be observed that the error obtained (with the experiment) did not have a linear relationship. The probable cause of the discrepancy between the results may be the measurement inaccuracies of the testing machine and the manufacturing inaccuracies. Moreover, it should be remembered that only linear models of the material were tested while observing a satisfactory convergence in the results obtained for small force values.

## 6. Conclusions

In this work, experimental results of blind rivet joints were presented. The experimental results were compared with the numerical analysis, and it was found that they complimented the analytical results. A detailed numerical stress analysis and plastic deformation analysis were performed on the joint connection. Additionally, linear and non-linear numerical material models were compared. The results obtained from these investigations led to the following conclusions:

- The selection of the material strength modeling method influences the results in the numerical strength analysis of the riveted joint in the entire load area (even for small values of the loading force, discrepancies in the results were observed);
- For small values of the force (about 10% of the load capacity of the riveted joint), too high values of elongation are observed in the numerical results, compared to the experimental ones (about 6% higher);
- The greatest difference in the deformation results was observed for the loads that comprise approximately 90% of the connection capacity. The highest difference was equal to 19.11%, and it was observed in the configuration of nonlinear materials. The linear model presents a smaller error (about 11%);
- It is assumed, by standards, that the allowable force should not exceed 311 N. It has been shown that, in this load range (from 0–30% of the maximum force transmitted by the riveted joint—1036 N), the selected material model does not have a significant impact on the numerical analysis results;
- An undervaluation of the maximum contact stress values (in the case of analytical pressure stress calculation) for higher values of the tensile force was observed;
- The stress values estimated using Hertz’s stresses were too high (20–40 times) compared to the results of the numerical analyses. These values, even for small load forces (e.g., 100 N), give values that exceed five times the tensile strength for the analyzed materials;
- The method of material modeling did not have a large impact on the maximum value of contact stresses obtained, as estimated by numerical methods.

As a result of the numerical analyses carried out and their reference to experimental studies, it can be concluded that the selection of linear models is sufficient for future numerical analyses. Such an assumption will affect the computation time and will allow for faster verification of future numerical analyses. However, it should be taken into account that, when modeling the crimping of the rivet and its subsequent loading, plastic deformation cannot be ignored. However, for the analysis of the axial tensile of the connection itself, taking into account the plasticity was not required. The presented research may be the basis for future calculations for critical load areas (above safety values), i.e., of aeroelastic issues.

The performed numerical tests and their reference to the experimental data may be the basis for future research focusing on the possibility of using relatively simple strength models (bilinear model instead of the multilinear model) in strength analyses, taking into account plasticization and deformation as a result of rivet clamping. Future studies could also focus on the range of forces closer to the joint breaking capacity (forces up to about 90% of the joint capacity have been considered in this publication).

## Figures and Tables

**Table 1:** Mechanical properties of the materials examined.

Aluminum Alloy	Young Modulus E, GPa	Poisson’s Ratio	Yield Stress Rp0.2, MPa	Ultimate Tensile Strength UTS, MPa
EN AW 2017A [27]	72	0.3	140	210
EN AW 5251 [28]	68	0.3	110	200

**Table 2:** Results of the analytical analysis of contact stress.

Force, N	Hertz Contact Stress, MPa	Pressure Stress (No Clearance), MPa
100	1092.7	24.3
200	1543.5	48.6
400	2182.8	97.3
600	2673.4	146
800	3086.9	194.6
900	3274.2	219

**Table 3:** Material properties of the bilinear material model.

Material Data	Aluminum Alloy EN AW 2017A (Sheet)	Aluminum Alloy EN AW 5251 (Rivet)
Density, kg/mm^3^	2700	2700
Young’s Modulus, GPa	72	68
Poisson’s ratio	0.3	0.3
Yield strength, MPa	210	140
Tangent modulus, MPa	1200	1000

**Table 4:** Comparison of the elongations of the riveted joint obtained numerically and experimentally, for different force values, with the percentage error to the experimental value.

Force F, N	Experiment	Linear-Linear	Linear-Linear	Nonlinear-Linear	Nonlinear-Linear	Nonlinear-Nonlinear	Nonlinear-Nonlinear
	Total Deformation Maximum						
100	0.03406 mm	0.0362 mm	106.27%	0.0361 mm	105.98%	0.0361 mm	105.98%
200	0.08766 mm	0.0731 mm	83.39%	0.0725 mm	82.71%	0.0725 mm	82.71%
600	0.25432 mm	0.2198 mm	86.42%	0.2182 mm	85.79%	0.2182 mm	85.79%
800	0.32101 mm	0.2924 mm	91.09%	0.2909 mm	90.62%	0.2911 mm	90.68%
900	0.36684 mm	0.3266 mm	89.04%	0.3005 mm	81.93%	0.2967 mm	80.89%

## Data Availability

Data is contained within the article.

## Appendix A

The following Appendix contains Table A1, Table A2, Table A3, Table A4 and Table A5 that summarize the results of the numerical strength analysis of a riveted joint and a comparison with the experimental results for the same joint.

Maximum Total Deformation and Maximum Equivalent Stress results are calculated for sheets only, but the rest of the results is directly calculated for rivet only. This approach to markings allows for a separate interpretation of the state of loads in the sheets and the rivet itself.

**Table A1:** Experimental and numerical results for the load force F = 100 N.

Experimental Work	Numerical Parameters	Linear-Linear	Nonlinear	
			Bilinear-Linear	Bilinear-Bilinear
displacement s, mm 0.034063	Maximum Total Deformation, mm	0.0362	0.0361	0.0361
	Maximum Equivalent Stress, MPa	54.9	54.9	54.9
	Maximum Total Deformation for rivet, mm	0.01982	0.0198	0.0198
	Maximum Equivalent Stress for rivet, MPa	50.0	50.3	50.3
	Maximum Shear Stress in rivet, MPa	11.6	11.6	11.6
	Minimum Shear Stress in rivet, MPa	−11.9	−12	−12

**Table A2:** Experimental and numerical results for the load force F = 200 N.

Experimental Work	Numerical Parameters	Linear-Linear	Nonlinear	
			Bilinear-Linear	Bilinear-Bilinear
displacement s, mm 0.08766	Maximum Total Deformation, mm	0.0731	0.0725	0.0725
	Maximum Equivalent Stress, MPa	108.4	114.7	114.7
	Maximum Total Deformation for rivet, mm	0.04004	0.0397	0.0397
	Maximum Equivalent Stress for rivet, MPa	98.1	99.2	99.2
	Maximum Shear Stress in rivet, MPa	22.6	25.1	25.1
	Minimum Shear Stress in rivet, MPa	−24.2	−25	−25

**Table A3:** Experimental and numerical results for the load force F = 600 N.

Experimental Work	Numerical Parameters	Linear-Linear	Nonlinear	
			Bilinear-Linear	Bilinear-Bilinear
displacement s, mm 0.254326	Maximum Total Deformation, mm	0.2198	0.2182	0.2182
	Maximum Equivalent Stress, MPa	388.8	403.5	238.3
	Maximum Total Deformation for rivet, mm	0.1202	0.1194	0.1194
	Maximum Equivalent Stress for rivet, MPa	313.4	146.6	146.6
	Maximum Shear Stress in rivet, MPa	111.9	82.3	82.3
	Minimum Shear Stress in rivet, MPa	−110.1	−81.5	−80.2

**Table A4:** Experimental and numerical results for the load force F = 800 N.

Experimental Work	Numerical Parameters	Linear-Linear	Nonlinear	
			Bilinear-Linear	Bilinear-Bilinear
displacement s, mm 0.321017	Maximum Total Deformation, mm	0.2924	0.2909	0.2911
	Maximum Equivalent Stress, MPa	548.3	531.2	230.1
	Maximum Total Deformation for rivet, mm	0.1598	0.1594	0.1596
	Maximum Equivalent Stress for rivet, MPa	474.6	151.7	153.4
	Maximum Shear Stress in rivet, MPa	158.6	83.4	82.2
	Minimum Shear Stress in rivet, MPa	−156.8	−83	−82.8

**Table A5:** Experimental and numerical results for the load force F = 900 N.

Experimental Work	Numerical Parameters	Linear-Linear	Nonlinear	
			Bilinear-Linear	Bilinear-Bilinear
displacement s, mm 0.3668414	Maximum Total Deformation, mm	0.32664	0.30055	0.29676
	Maximum Equivalent Stress, MPa	805	577.3	231.3
	Maximum Total Deformation for rivet, mm	0.17856	0.16471	0.16272
	Maximum Equivalent Stress for rivet, MPa	721.2	164.3	166.9
	Maximum Shear Stress in rivet, MPa	176.5	84.1	83.4
	Minimum Shear Stress in rivet, MPa	−176.1	−84	−83.3
